# American Medical Association

**Published:** 1878-07

**Authors:** 


### Editorial.

---

We devote our entire editorial space to the reports of the American Medical Association and Association of Medical Colleges, at Buffalo. It will be seen that the meetings of both for the year 1879 will be held in Atlanta, and we hope that the medical profession, not only in Georgia, but the whole South, will take a lively interest in the meeting here, commencing on the first Tuesday in May next.

---

AMERICAN MEDICAL ASSOCIATION.—The opening session of the twenty-ninth annual meeting of the American Medical Association was held at Buffalo, New York, Tuesday, June 4, 1878. The Association was called to order at 11 A. M., by the President, Dr. T. G. Richardson, of New Orleans. Prayer was offered, when the address of welcome was delivered by Dr. Thomas F. Rochester, of Buffalo. Prof. James P. White introduced President T. G. Richardson, of Louisiana, who delivered his annual address.

Professors White and Gross paid high compliments to the author of the address.

The speaker and his four predecessors were appointed to suggest means for carrying out the recommendations made.

Dr. Wm. Brodie, of Detroit, then read his report as a delegate to the Canadian Medical Association.

The report was referred to the Committee of Publication.

Dr. L. A. Sayre, of New York, made a verbal report of his visit as a delegate to the British Medical Association.

After some announcements, the Association adjourned.

---

*Wednesday, June 5th.*—The meeting was called to order by the President.

The following report was then offered by the Judicial Council:

The charges against Dr. J. M. Keller, presented by Dr.

G. W. Lawrence, were dismissed as being unworthy of any consideration.

The charges against the Society of Grand Rapids, Michigan, presented by Dr. P. J. Dwyer, are incomplete and unaccompanied by witnesses and testimony whereby the Judicial Council might be able to act.

The Arkansas petitions referring to the State Medical Society were dismissed without action.

The charges against the Michigan State Society, by Dr. W. W. Jones, will be reported upon by Dr. N. S. Davis, Chairman of the Judicial Council.

The charges against W. F. Barr, M.D., of Abingdon, Va., presented by Dr. Ulrich, of Chester, Pa., were dismissed by the Council.

With reference to the communication from the State Medical Society of Arkansas, notifying the Association that the Hot Springs and Garland County Medical Society was not recognized by the State Medical Society, and protesting against the reception of any member of that Society, it is decided that, under the by-laws of this Association, said Hot Springs and Garland County Society loses its recognition with the Association from date of its severance from the State Society.

Professor Davis then read the appended report of the Judical Council concerning the charges against the Michigan State Medical Society, referred by the Association at the annual meeting in June, 1877:

The charge in this case was alleged violation of the code of ethics on the part of the Michigan State Medical Society in electing as a delegate to this Association Dr. E. L. Dunster, of the University of Michigan, knowing him to be engaged in aiding and abetting the graduation of students devoted to an exclusive dogma in medicine.

After a most careful examination of the code of ethics, as it has appeared in the transactions of this Association from year to year, the Judicial Council fail to find any section or paragraph in it that refers, even remotely, to the practice that constitutes the foundation of the charge under consideration. That any member of the medical profession proper should ever engage in teaching, examining and certifying to the qualifications of students, knowing that such teaching and examinations were to aid said student in obtaining a diploma directly admitting them into a few—a fraternity of irregular practitioners—was evidently not contemplated by the framers of our Code of Ethics; and hence they inserted no clause or section bearing upon that subject. The only provision in the Code referring to those engaged in an attempt to practice medicine in accordance with some "exclusive dogma," is in the section regulating consultations at the bedside of the sick. If the Judicial Council of this national organization should assume that the section of the Code just referred to indicated the "spirit" of those who framed and adopted it, and on that assumption apply it to matters and practices entirely foreign to those mentioned in the doctrine itself, it would not only violate all the accepted principles of judicial construction, but would establish a precedent in latitudinous construction of the Ethical Code more dangerous to the best interests of the profession than all the evils sought to be remedied in the case under consideration.

It is true that this Association has adopted at different times two resolutions having reference to the subject involved in the Michigan State Medical Society, which still stand as expressions of opinion unrepealed. But these resolutions constitute no part of the Code of Ethics; neither is obedience to them enjoined by the constitution and by-laws on State and local medical societies as a condition of representation in this Association. Therefore, while deprecating the practice of aiding or abetting in any way the teaching and graduation of students known to be supporters of irregular and exclusive dogmas in medicine, as beneath the dignity of right-minded teachers of an honorable and liberal profession, your Judicial Council can find no clause in either the Constitution, By-Laws, or Code of Ethics, as they now exist, under which the charge against the Michigan State Medical Society can be entertained and adjudicated.

A discussion followed the reading of this report by Drs. J. M. Toner, N. S. Davis, S. C. Bussey, J. J. Woodward, Wm. Brodie, and Thomas Menees.

Professor Henry H. Jones, of Philadelphia, Chairman of the Section of Surgery and Anatomy, then delivered his address, taking for his subject, Certain Points on the Pathology of the Bones, especially Tubercle.

Dr. Moses Gunn, Vice President, took the chair, and Dr. E. W. Jenks, of Detroit, Michigan, Chairman of the Section on Obstetrics and Diseases of Women and Children, delivered an address on The Causes of the Sudden Death of Puerperal Women. It was referred to the Section on Obstetrics for publication.

Dr. J. H. Bronson, of Massachusetts, offered the following preamble and resolution:

WHEREAS, By the report of the Judicial Council, submitted this day, we are informed that the Ethical Code of this Association is imperfect, in that it does not recognize by its letter a conceded violation of the spirit of our profession in its relation to irregular medicine, therefore,

*Resolved*, That said Council be instructed to submit to this Association, at their meeting, for its consideration, an amendment to the Code covering this omission.

The above was, on motion, referred to all of the members of the Judicial Council as a committee.

Dr. Foster Pratt, of Kalamazoo, Michigan, offered a resolution in relation to the legal status of the insane, which was referred to the Section on Medical Jurisprudence.

---

*Thursday, June 6th.*—The general session opened with President Richardson in the chair.

Dr. N. S. Davis, of the Judicial Council, presented the following report, proposing an amendment and addition to the Code of Ethics, and action on the same was deferred, according to the rules, for one year:

In obedience to the instructions of this Association, the Judicial Council, acting in the capacity of a committee, have unanimously instructed me to report to your honorable body the following amendment and addition to Paragraph I, Article I, of the second division of the Code of Ethics, under the general heading, "Of the duties of Physicians to each other, and to the Profession at large," and the special heading, "Duties for the Support of Professional Character." The same, when finally adopted, to be added at the end, and to constitute a part of said Paragraph I, of Article I. The proposed addition is in these words: "And hence, it is considered derogatory to the interests of the public and the honor of the profession for any physician or teacher to aid, in any way, the medical teaching or graduation of persons, knowing them to be supporters and intended practitioners of some irregular and exclusive system of medicine."

Dr. J. M. Toner, of Washington, D. C., Chairman of the Committee on Necrology, presented his annual report. It was referred at once to the Committee on Publication.

Dr. A. L. Loomis, of New York, Chairman of the Section on Practical Medicine, Materia Medica, and Physiology, was then introduced, and delivered an able address. He noticed some of the important advances made in practical medicine, physiology, and materia medica, and also discussed at length the climatic treatment of pulmonary phthisis.

Dr. Toner, of the Committee on Nominations, announced that they were ready to report. Accordingly, Dr. Hupp, of West Virginia, read the following, which was, on motion of Dr. White, of Buffalo, accepted and adopted unanimously:

After due consideration, the Committe on Nominations respectfufly report that they have nominated the following gentlemen for the various offices named, to-wit:

*President.*—Theophilus Parvin, M.D., of Indiana.

*Vice-Presidents.*—A. J. Fuller, M.D., of Maine; W. F. Westmoreland, M.D., of Georgia; John Morris, M.D., of Maryland; John H. Murphy, M.D., of Minnesota.

*Treasurer.*—Richard Dunglison, M.D., of Pennsylvania.

*Committee on Library.*—J. Eliot, M.D., of District of Columbia.

*Assistant Secretary.*—Scott Todd, M.D., of Atlanta, Ga.

*Committee of Arrangements.*—J. P. Logan, Chairman; H. V. M. Miller, G. G. Crawford, H. L. Wilson, J. F. Alexander, J. M. Johnson, Charles Pinckney, V. H. Taliaferro, J. T. Johnson, of Atlanta, Ga.

*Committee on Prize Essays.*—Robert Battey, of Rome, Ga.;

J. G. Westmoreland, Wm. A. Love, Robert Ridley, of Atlanta, Ga.; Henry F. Campbell, of Augusta, Ga.; J. H. Van Deman, of Chattanooga, Tenn.

Next place of meeting, Atlanta, Ga.

Time of meeting, the first Tuesday in May, 1879.

Dr. J. L. Cabel, of the University of Virginia, Chairman of the Section on State Medicine and Public Hygiene, delivered an address.

On motion, the address of Dr. Cabel was referred to the section on Hygiene.

Dr. Sayre, of New York, then rose to a question of personal privilege, and asked that the Secretary be requested to place him on record as opposed to the resolution adopted at Detroit last year, which declared that a fracture of all long bones could not occur without shortening. The request was granted.

---

*Friday, June 7th.*—Dr. J. S. Hibbard, of Indiana, offered the following resolution, which was adopted:

*Resolved*, That hereafter it shall be the duty of the Committee on Necrology to confine their reports to the death of medical men who have been members of this Association, and at the time of death were still in good fellowship, or honorably separated from the Association.

Dr. A. N. Bell moved the continuance of the committee appointed two years ago, for the organization of State board of health. Carried.

The President announced the following delegates:

To European Medical Societies—Drs. Sims, Drysdale, Seguin, Daly, Halberstadt, Levis and Pancoast.

To the Canadian Medical Association—Drs. Brodie, Todd, E. N. Brush and W. Clarke.

Dr. X. C. Scott called up the resolution of last year, creating a new Section of Ophthalmology, Otology and Laryngology, to be known as Section VI, and moved its adoption. Carried.

On motion of Dr. E. Smith, of Detroit, Dr. H. Knapp, of New York, was made chairman of the new Section, and X. C. Scott, of Ohio, was made secretary.

The minutes of Sections II and V were received and referred to the Committee of Publication.

The Secretary read the annual report of the Treasurer, Richard J. Dunglison, of Philadelphia.

The report was adopted and referred to the Publication Committee.

On motion of Dr. Davis, the Treasurer was instructed to pay the sum of $700 as a honorarium to the permanent Secretary for his services.

The following report of the Committee on Prize Essays was read by the local Secretary and adopted:

Your Committee, to determine the merits of prize essays, would respectfully report: That they have had three separate papers submitted to their inspection. Two of these papers present subjects of very great interest, and show original researches, but are too imperfect, in the estimation of the Committee, to command a prize. The remaining paper, in the judgment of your Committee, is fully up to the requirements. Indeed, the paper is so elaborate as to fill a large space in the volume of the Transactions of the Association. The paper should be considered as *two* and not as *one.* The analysis of 789 cases of operation on the carotid artery, and the careful and minute measurements of the artery and its branches in one hundred and twenty-one subjects, showing the range of variation and the per centage of the same, followed by inferences, bold and original, naturally constitutes a paper complete in itself. Another one on the same plan, with reference to the innominata and subclavian, being an analysis of 300 cases, and the observation of fifty-two subjects, is presented to us in such a manner that we may consider the whole as one prize, or they may compete for both.

Your Committee believe that both prizes should be awarded to the two essays by one person. The motto is, "*Tempora mutantur et nos matumur in illis.*"

E. H. Moore, *Chairman.*
Thos. Lothrop,
H. R. Hopkins,
W. W. Miner.

*Buffalo, N. Y., June 6th,* 1878.

The recommendation of the committee was unanimously adopted.

On opening the sealed envelope, the name of the successful essayist was found to be Dr. John A. Wyeth, of New York City, and the announcement was received with applause.

Dr. J. M. Kellar, of Arkansas, offered the following, which was laid over for one year:

*Resolved*, That in the future the Committee on Nominations shall present the name of no person for appointment or election to office or position, save on the Committees on Necrology and Climatology, unless the party nominated be in attendance on the Association at the time.

Dr. J. J. Caldwell, of Maryland, offered a resolution creating a new Section upon "Neurology and Electrology." Action was deferred for one year.

Dr. Maddux, of Maryland, offered a resolution creating a Section on "Diseases of the Genito-Urinary Organs, including Dermatology and Syphilis." Laid over.

Dr. J. M. Toner offered a resolution in relation to the late Prof. Joseph Henry, of the Smithsonian Institute.

Dr. Parvin, the President-elect, introduced by President Richardson with a few complimentary remarks, returned his thanks for the honor conferred in eloquent and fitting terms.

Resolutions of thanks were then passed, and the Association adjourned.

